# Impulsive people have a compulsion for immediate gratification—certain or uncertain

**DOI:** 10.3389/fpsyg.2015.00515

**Published:** 2015-05-05

**Authors:** Wojciech Białaszek, Maciej Gaik, Elton McGoun, Piotr Zielonka

**Affiliations:** ^1^Department of Behavior Analysis, University of Social Sciences and Humanities, Warsaw, Poland; ^2^Department of Economic Psychology, Kozminski University, Warsaw, Poland; ^3^School of Management, Bucknell University, Lewisburg, PA, USA; ^4^Department of Econophysics, Warsaw University of Life Sciences, Warsaw, Poland

**Keywords:** impulsivity, delay discounting, probability discounting, time-probability trade-off, magnitude effect, sign effect

## Abstract

Impulsivity has been defined as choosing the smaller more immediate reward over a larger more delayed reward. The purpose of this research was to gain a deeper understanding of the mental processes involved in the decision making. We examined participants’ rates of delay discounting and probability discounting to determine their correlation with time-probability trade-offs. To establish the time-probability trade-off rate, participants adjusted a risky, immediate payoff to a delayed, certain payoff. In effect, this yielded a probability equivalent of waiting time. We found a strong, positive correlation between delay discount rates and the time-probability trade-offs. This means that impulsive people have a compulsion for immediate gratification, independent of whether the immediate reward is certain or uncertain. Thus, they seem not to be concerned with risk but rather with time.

## Introduction

Perhaps the two most fundamental principles in financial decision making are the time value of money and the trade-off between risk and return. It is not so surprising, then, that in the laboratory, numerous experiments have attempted to measure how people make the trade-offs between reward now and reward later and trade-offs between reward that will definitely be received and reward that might or might not happen (for a review see: Green and Myerson, 2004, 2010; McKerchar and Renda, 2012). Real life decisions, however, are not so simple, usually involving both temporal and probabilistic elements. That is, reward might be received at a variety of points in time with varying degrees of certainty for each. How these more complex decisions are made, then, is of considerable practical interest.

When offered a choice between two payoffs, people usually prefer a larger payoff to a smaller one, an earlier payoff to a later one, and a certain payoff to an uncertain one. In combination, those who prefer a smaller, earlier payoff to a larger, later one are said to be more impulsive (Logue, 1988); those who prefer a smaller, certain payoff to a larger, uncertain one are said to be more risk averse (Shead and Hodgins, 2009). Delay discounting is the decrease in the subjective present value of an outcome according to the hyperbolic function (Mazur, 1987, 1988): The greater the impulsivity, the higher the discount rate. Probability discounting is the decrease in the subjective value of an outcome as a function of its likelihood: The greater the risk aversion, the higher the discount rate. Discount rates are related to the amount of the outcome. Green et al. (1997) found a magnitude effect for temporal discounting; the discount rate decreased as the amount of the delayed payoff increased. Green et al. (1999) found a reverse-magnitude effect for probability discounting; the discount rate increased as the uncertain payoff increased. Discount rates are also related to the sign of the outcome, being smaller for losses than for gains for both temporal discounting (Baker et al., 2003; Murphy et al., 2001) and probability discounting (Estle et al., 2006; Mitchell and Wilson, 2010). The sign effect for delay discounting means that people express a tendency to take gains quickly but to postpone losses. The sign effect for probability discounting means a greater risk aversion for gains than for losses, that is, a tendency to lock in gains but to let potential losses ride, hoping they will not occur.

Is impulsivity independent of individual’s risk aversion, that is, is it purely a consequence of her desire for immediate gratification? Or is impulsivity at least somewhat correlating with risk aversion. Thus far, empirical tests have not been conclusive, with researchers finding no correlation or weakly positive correlation between impulsivity and risk aversion (Myerson et al., 2003; Ohmura et al., 2006; Shead and Hodgins, 2009).

Do people choose smaller, immediate reward because they are just reluctant to wait, compelled to get a reward instantly (even in a form of a lottery)? In other words: what is the nature of impulsivity. Very little research has examined the mental trade-off between the delay of a certain payoff and the probability of an uncertain, immediate one. Rachlin et al. (1991) have studied people’s delay and probability discount functions and combined them into a time-probability trade-off function. They then showed that the probability discount function can be derived from the delay discount function. In this paper we approach the subject more directly by measuring a person’s time-probability trade-off—the risk a person is willing to assume in order to get an immediate payoff instead of waiting for it—thereby clarifying the foundations of impulsivity.

We examined the correlations between the time-probability trade-off [*p*(*t*)] and delay [*f*(*t*)] and probability [*f*(*p*)] discount rates. In short, our research hypothesis is the following: Impulsive people choose smaller, immediate reward over larger, delayed ones. They have a need for immediate gratification, even if the immediate reward is uncertain. If this hypothesis is correct, there will be a positive correlation between the delay discount rate and the time-probability trade-off.

## Materials and Methods

### Participants

Two hundred and seventeen participants (91 males and 126 females), ranging in age from 19 to 23 years and enrolled in obligatory lectures, were recruited for the study. They were all students of the Warsaw University of Life Sciences. All participants signed informed consent forms, and the procedures were approved by the local ethics committee (Kozminski University).

### Data Analysis

Of 217 individuals who participated, data for 12 were not considered due to non-systematic discounting. Discounting data were considered systematic and used if: (a) the participant had a higher initial indifference point (example 95% chance of obtaining PLN 200 or obtaining PLN 200 in 6 months) than the final indifference point (a 5% probability or a 5 years delay). (This criterion assumes delay decreases the value of a reward); (b) the participant’s indifference points did not increase across consecutive delays (or probabilities) by more than 20% of the larger later or larger more probable reward. (Substantial increases in the value of a reward across delays or probabilities suggests that the value of a reward is enhanced with increased delay or risk); (c) in both conditions—the probability and delay discounting components of the procedure—the data met criterion (a) and (b). These criteria are based on the expectation of a monotonically decreasing discounting function and are similar to the algorithm used by Johnson and Bickel (2008). We did not make any assumptions regarding the components of the time and probability trade-off, because there has been no previous research on this topic establishing any precedents. To check the magnitude effects and gain/loss asymmetry, we conducted three two-way ANOVAs separately for *p*(*t*) (time-probability trade-off) conditions, *f*(*t*) (delay discounting) and *f*(*p*) (probability discounting).

### Measures

As the measures of the rates of delay and probability discounting and the measure of the time-probability trade-off, we used simple arithmetic means computed from all indifference points in each condition. These measures are very similar to those of the area under the curve (AUC, Myerson et al., 2001), and we confirmed that the simple means highly correlated with the AUC measures (for probability: *r* = 0.989; *p* < 0.001; and for delay *r* = 0.993; *p* < 0.001). We have used this measure to maintain consistency for dependent variable measures across conditions. The means are directly comparable and meaningful, that is, showing directly how much trade-off occurs between probability and time. Indifference points in probability and delay discounting were expressed as a ratio of the adjusted reward and uncertain/delayed reward. For the time-probability trade-off, the dependent variable was probability.

### Procedure

There were four analogous, independent between-group conditions in this study. Each participant was randomly assigned to one of the four of them, either a gain or a loss of PLN 200 (respectively, *N* = 50 and *N* = 55), or a gain or a loss of PLN 5000 (respectively, *N* = 57 and *N* = 43). At the time of the study the value of these reward was around USD 70 and USD 1700. All monetary amounts were presented in Polish Zloty national currency (PLN).

Participants made choices between two alternatives presented to them on their computer screens by clicking on their preferences. Each participant went through three parts of the experimental procedure addressing: (1) the time-probability trade-off *p*(*t*), (2) delay discounting *f*(*t*), and (3) probability discounting *f*(*p*). The order of within-subject conditions was counterbalanced.

For every condition, the algorithm of the procedure was based on the same adjusting gain or loss algorithm, adapted from the procedure by Du et al. (2002). For the time-probability trade-off, the probability of immediate reward was adjusted. For the delay discounting and the probability discounting, the immediate values or the certain values of the reward respectively were adjusted according to participant’s choices. So for each within-subject condition, four indifference points were obtained.

For example, to measure *p*(*t*) in one condition, participants were faced with a first choice of receiving PLN 200 with a 50% chance or PLN 200 delayed by 6 months. In this condition the amounts of reward were equal, since we wanted to measure only the time-probability trade-off effect. In consecutive steps the odds of winning the lottery were increased or decreased in subsequent trials based on the participant’s previous response. If a lottery ticket was selected, the probability of winning the lottery was decreased, if the delayed payment was selected, the lottery odds were increased. The magnitude of change after the first choice was 25%, which was increased or decreased by half of the previous magnitude in consecutive steps. Therefore, if the delayed option was chosen, the next choice would be between PLN 200 with a 75% chance of receiving and PLN 200 in 6 months. The algorithm was set to six choices per delay. In this condition there were four delays: 6 months, 12 months, 3 years and 5 years. The loss condition was analogous to that of the gain, the only change being in the direction of adjustment. When the lottery was chosen, its value in the next step increased, and when the delayed option was chosen, the probability of the lottery decreased.

With the two other conditions, *f*(*t*) and *f*(*p*), the rules were the same. For delay discounting, participants made choices between smaller, immediate gains or losses and a delayed amount. The gain (or loss) was delayed by 6 months, 12 months, 3 and 5 years. The smaller amount was always set at the beginning to the half of the larger payment. For probability discounting, the chances of receiving the reward were presented in percentages and were set to 95, 70, 30, and 5%. When the immediate (or certain) gain was chosen, its value decreased by half, and when the delayed (or risky) option was chosen, the alternative increased. As in the other parts, the same happened in the loss condition, but the changes were opposite, making the immediate (or certain) alternative less attractive when it was chosen.

## Results

We conducted analyses in two steps. In the first step, we analyzed correlations among the time-probability trade-off, delay discounting, and probability discounting. In the second step, we used two factor ANOVA analysis to investigate whether factors (amount and sign) affected the height of indifference points. All pairwise, multiple comparisons used Sidak’s correction.

The results of the correlation analyses showed that there is a strong relationship between *f*(*t*) and *p*(*t*), which confirms our hypothesis. There is also a positive relationship between delay and probability discounting for small payments, but for large payments this relationship is not significant. The relationship between the time-probability trade-off and probability discounting was not significant. These results are presented in Table 1.

**TABLE 1:** **Pearson’s r correlation coefficients for *p*(*t*), *f*(*t*), and *f*(*t*)**.

		PLN 200		PLN 5000	
		*f*(*t*)	*f*(*p*)	*f*(*t*)	*f*(*p*)
Gains	*p*(*t*)	0.578*	0.077	0.694*	0.146
	*f*(*t*)	1	0.464*	1	0.241
Losses	*p*(*t*)	0.544*	0.199	0.681*	0.146
	*f*(*t*)	1	0.435**	1	0.168

*p < 0.001; **p < 0.05; otherwise—non-significant.

**FIGURE 1:**
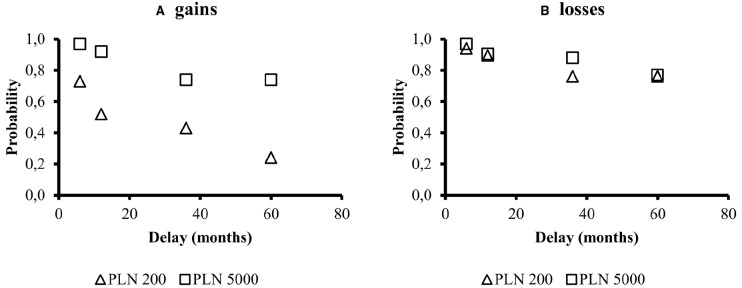
**Median indifference points for various magnitudes (PLN 200 and PLN 5000) and signs of an outcomes (panel A: gains panel B: losses) in the time-probability trade-off [*p*(*t*)].** The indifference points represent an equilibrium between delay and probability: the subjective value of a delay is expressed as probability of outcome occurrence.

**FIGURE 2:**
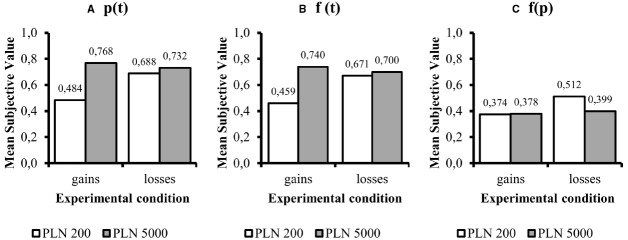
**Mean subjective value expressed as averaged indifference points.** All data are expressed as proportions. In case of time-probability trade-off the means can be interpreted in terms of probability, and in *f*(*t*) and *f*(*p*) conditions as a proportion of immediate/certain subjective value to larger later/certain reward. The panels refer to (standard deviations are presented in order gain PLN 200, gain PLN 5000, loss PLN 200, loss PLN 5000): **(A)** *p*(*t*)—probability-time trade-off (SDs respectively: 0.245, 0.205, 0.301, 0.254); **(B)** *f*(*t*)—delay discounting (SDs respectively: 0.236, 0.250, 0.289, 0.288); **(C)** *f*(*p*)—probability discounting conditions (SDs respectively: 0.181, 0.196, 0.225, 0.253).

Figure 1 illustrates the trade-off between time and probability. The same data is also shown on Figure 2A, but averaged across indifference points from all condition. We performed all statistical analyses on averaged indifference points across probabilities or delays in order to fulfill all of the assumptions of parametric factorial data analytic methods.

One factor was amount (PLN 200 and PLN 5000) and the other was sign (gain or loss). In the *p*(*t*) condition there was a significant main effect of amount [*F*(1,201) = 21.288; *p* < 0.001; η^2^ = 0.096] and of sign [*F*(1,201) = 5.592; *p* = 0.019; η^2^ = 0.027]. The interaction effect was also significant [*F*(1,201) = 11.421; *p* < 0.001; η^2^ = 0.054]. For interaction there were significant differences between the two amounts for gains, but not for losses (respectively, *p* < 0.001 and *p* = 0.395). There were also significant differences in the means of gains and losses, but only in the PLN 200 condition (*p* < 0.001) and not in the PLN 5000 condition (*p* = 0.481). The descriptive statistics for p(t) conditions are presented on Figure 2A.

For delay discounting, there was a significant main effect of sign [*F*(1,201) = 5.318; *p* = 0.022; η^2^ = 0.026] and of amount [*F*(1,201) = 17.044; *p* < 0.001; η^2^ = 0.078]. The interaction effect was also significant [*F*(1,201) = 11.303; *p* < 0.001; η^2^ = 0.053]. Delay discount rates displayed he same relations as for *p*(*t*). Only for smaller amounts were losses discounted slower than gains (*p* < 0.001). For PLN 5000 these differences were insignificant (*p* = 0.464). In the gains conditions larger reward were discounted slower than smaller ones (*p* < 0.001). There was no magnitude effect in loss conditions (*p* = 0.597). The descriptive statistics for f(t) conditions are presented on Figure 2B.

Probability discount rates showed significant effects for sign [*F*(1,201) = 6.954; *p* = 0.009; η^2^ = 0.033] but not for amount [*F*(1,201) = 3.252; *p* = 0.073; η^2^ = 0.016]. There was no significant interaction between the two factors [*F*(1,201) = 3.818; *p* = 0.052; η^2^ = 0.019]. For probability discount rates, losses were discounted less steeply than gains. Although neither the main effect, nor interaction reach the statistical significance criterion, the result can be classified as statistical tendency. The descriptive statistics for f(p) conditions are presented on Figure 2C.

The above analyses show a magnitude effect for *p*(*t*) and *f*(*t*), but a reverse magnitude effect was absent in *f*(*p*). There was a sign effect for the time-probability trade-off and delay discounting, but only for small amounts. All descriptive statistics for these comparisons are presented in Figure 1 (standard deviations are displayed in Figure 1 captions).

## Discussion

The primary goal of the research was to examine the nature of impulsivity. If one considers the familiar connotations of the label, one might expect impulsive individuals to not only be unable to postpone reward, but also to be risk takers. This would imply a negative correlation between probability and delay discounting, i.e., participants would choose smaller sooner reward instead of larger delayed one and riskier, larger option instead of smaller, but certain. However, prior research (Myerson et al., 2003; Estle et al., 2007; Green and Myerson, 2013) has shown that the correlation is slightly positive, meaning that impulsive individuals not only choose smaller, immediate reward rather than larger, later ones, but they are also more risk averse than individuals who choose larger, later reward. Our results are consistent with this. For small amounts (both gains and losses) the delay discount rate is significantly positively correlated with the steepness of probability discounting. This means that impulsive individuals tend to be less risk prone, and self-controlled people tend to be more risk prone (for small payoffs). Then, the crucial problem is the following: do impulsive individuals choose a smaller, immediate reward, regardless if the reward is certain or not, because they are reluctant to wait, compelled to get a reward instantly? In order to answer this question, we examined the correlation between delay discounting and the time-probability trade-off. Our research hypothesis was that impulsive people will choose smaller, immediate reward over larger, delayed ones because they have a need for immediate gratification, even if the immediate reward is uncertain. In other words, there will be a positive correlation between the delay discount rate and the time-probability trade-off.

The experimental design enabled us to study delay discounting and the time-probability trade-off, taking into accounting both the amount effect and the sign effect. For all amounts and signs, the time-probability trade-off rate was significantly positively correlated with the delay discounting rate. Therefore, when confronted with an uncertain outcome now and a certain outcome in the future, people tend to assess the situation as if they were simply confronting a small amount now versus a larger amount in the future, regardless of amounts and signs. Impulsive people prefer smaller, immediate reward over larger, delayed ones because they have a need for immediate, even uncertain, gratification. A revealed time-probability trade-off rate shows how much risk an individual is willing to bear in order to receive a reward immediately rather than waiting. Here, the main result of our research is a confirmation of the research hypothesis, finding a strong positive correlation between the delay discounting rate and the time-probability trade-off. This means that impulsive people choose smaller, immediate reward over larger, delayed ones because they are motivated by a desire for instant gratification, even if that means bearing the uncertainty of a lottery. The risk inherent in delay may not be a factor in their decisions. People with self-control having the ability to wait will choose larger, later reward. Moreover, the time-probability trade-off is uncorrelated with the probability discounting. This means that the question “How much is one able to risk in order not to wait?” has nothing common with the question “How much is one able to risk?” The classical risk attitude describes human preferences when all options are available in the present without the component of delay. It seems that when one of the outcomes is immediate, an individual tends to act not according to her risk attitude but according to her time-probability trade-off rate. The time-probability tradeoff measures the conversion of delay into risk, since one of the outcomes is certain and delayed and the other is immediate and uncertain.

There are other findings which go beyond previous research. For the time-probability trade-off, our results showed a sign effect for small amounts (losses have larger subjective value than gains) and an amount effect for gains (bigger payoffs have larger subjective values than smaller ones).

There are other findings which confirm previous research. These include: (1) The sign effect for delay discounting for small amounts (Thaler, 1981; Estle et al., 2006) and for probability discounting for large and small amounts taken together, which is consistent with the predictions of prospect theory (Kahneman and Tversky, 1979; Tversky and Kahneman, 1981). This means people prefer to realize gains immediately and postpone losses (if they happen at all) until the future. (2) Small gains were discounted faster than large gains for the time-probability trade-off and for delay discounting. This means that people want to realize a small gain immediately but are willing to wait for a big score. For the time-probability trade-off, this means that they do not want to risk so much in case of large payoffs, which is a new result. For probability discounting the main effect of amount was not significant. The interaction of amount and sign of the outcome was not significant. Both have p values bordering significance which might imply the statistical tendency. We can see a tendency toward a reversed magnitude effect in the domain of losses. As noted by Estle et al. (2006) in the domain of losses the effects of amount if present are rather small and not always reliable.

The present research was done on hypothetical, not real reward. It has been shown that the discounting process is comparable across real and hypothetical payments (Johnson and Bickel, 2002; Madden et al., 2003; Lawyer et al., 2011; Matusiewicz et al., 2013).

Overall, the results suggest that when one of the outcomes is immediate, an individual tends to act not according to her risk attitude, which matters for instant outcomes but according to her time-probability trade-off rate. A strong positive correlation between the time-probability trade-off and the delay discounting rate indicates that impulsive people choose smaller, immediate reward over larger, delayed ones because they may be mainly motivated by a desire for instant gratification, even if that means bearing the uncertainty of a lottery.

## Author Contributions

All authors contributed to the presented work. Each of the authors took part in drafting or revising it critically for important intellectual content and approved the final version to be published. Also all authors ensured that questions related to the accuracy or integrity of any part of the work are appropriately investigated and resolved. Especially: WB, PZ: substantial contribution to the conception and design of the work, acquisition, analysis, interpretation of data for the work; MG: acquisition of the data; EMG: interpretation of data.

### Conflict of Interest Statement

The authors declare that the research was conducted in the absence of any commercial or financial relationships that could be construed as a potential conflict of interest.
